# Engineering of a Bacterial Biosensor for the Detection of Chlorate in Food

**DOI:** 10.3390/bios13060629

**Published:** 2023-06-06

**Authors:** Alexandra Vergnes, Jérôme Becam, Laurent Loiseau, Benjamin Ezraty

**Affiliations:** Aix-Marseille University, CNRS, Laboratoire de Chimie Bactérienne, Institut de Microbiologie de la Méditerranée, 13009 Marseille, France; vergnesalexe@gmail.com (A.V.); jbecam@imm.cnrs.fr (J.B.); loiseau@imm.cnrs.fr (L.L.)

**Keywords:** bacteria biosensor, chlorate detection, *Escherichia coli*, methionine sulfoxide reductase

## Abstract

Chlorate can contaminate food due to the use of chlorinated water for processing or equipment disinfection. Chronic exposure to chlorate in food and drinking water is a potential health concern. The current methods for detecting chlorate in liquids and foods are expensive and not easily accessible to all laboratories, highlighting an urgent need for a simple and cost-effective method. The discovery of the adaptation mechanism of *Escherichia coli* to chlorate stress, which involves the production of the periplasmic Methionine Sulfoxide Reductase (MsrP), prompted us to use an *E. coli* strain with an *msrP-lacZ* fusion as a biosensor for detecting chlorate. Our study aimed to optimize the bacterial biosensor’s sensitivity and efficiency to detect chlorate in various food samples using synthetic biology and adapted growth conditions. Our results demonstrate successful biosensor enhancement and provide proof of concept for detecting chlorate in food samples.

## 1. Introduction

Exposure to chlorate (ClO_3_^−^) in food and in drinking water over a long period of time is a potential health concern, particularly for children with mild-to-moderate iodine deficiency [1,2]. Although the total intake in a single day is unlikely to exceed the recommended level for consumers in all age groups, chronic exposure can inhibit iodine absorption. Chlorate can be present in food due to the use of chlorinated water for food processing or in the disinfection of food processing equipment. Drinking water is the primary source of chlorate in the diet, contributing to up to 60% of chronic chlorate exposure in children. The European Food Safety Authority (EFSA) has set a tolerable daily intake (TDI) of 3 µg/kg body weight per day for long-term exposure to chlorate in food [1]. In addition, acute exposure to high levels of chlorate over a single day can be toxic to humans, leading to kidney failure. The EFSA has recommended a safe intake level for a daily intake of chlorate (called the “acute reference dose”) of 36 µg/kg body weight per day [1]. There is a need for a simple and cost-effective method to determine and quantify chlorate in liquids and foods [3]. Methods such as chromatography, mass spectrometry, and Raman spectrometry have been developed for (per)chlorate detection, offering increased sensitivity and accuracy. Although, these techniques have been successfully applied to a wide range of environmental samples, demonstrating exceptional accuracy in identifying (per)chlorate concentrations even in complex matrices, they suffer from limitations, as they are labor-intensive, expensive, and time-consuming, and require highly trained personnel [4,5]. Consequently, they are unsuitable for the rapid delineation of contaminated samples. Approximately ten years ago, the concept of utilizing a detection methodology based on biosensors or bioassays emerged as a potential solution to these challenges. The Coates lab has made significant advancements in this area by developing an efficient bioassay for (per)chlorate detection [6]. Their approach involves combining purified perchlorate reductase from *Dechloromonas agitate* with an enzymatic colorimetric assay. More recently, the use of genetically modified organisms, such as the nematode *Caenorhabditis elegans* or the bacteria *Escherichia coli*, as biosensors for (per)chlorate detection has emerged [7,8]. Unfortunately, the detection threshold of these biosensors is too high to be practical or effective for use.

Chlorate has long been recognized as a potential threat to bacteria due to its conversion into the toxic chlorite (ClO_2_^−^) in anaerobic conditions via reduction by nitrate reductase [9,10,11,12]. Despite this knowledge, the precise molecular mechanism underlying chlorate toxicity has only recently been uncovered. Recent studies have revealed that chlorite oxidizes the methionine residues of proteins, highlighting the critical role of methionine-reducing enzymes, specifically the Methionine Sulfoxide Reductases (MSRs) [13,14], in conferring resistance to chlorate/chlorite stress in bacteria [15,16]. In the case of *E. coli*, it has been demonstrated that MsrP, the periplasmic MSR, is overproduced during chlorate stress in order to repair periplasmic oxidized proteins [15]. The discovery of the bacterial adaptation mechanism to chlorate stress prompted us to employ an *E. coli* strain containing an *msrP-lacZ* fusion as a biosensor for detecting chlorate in commercial growth media for bacteriology. Through this approach, we were able to identify the presence of chlorate in some batches of Casamino Acids and agar [17]. The objective of this study was to enhance the sensitivity and efficiency of the bacterial chlorate biosensor by employing synthetic biology and optimized growth conditions of the strain. This would enable us to evaluate the biosensor’s performance for detecting chlorate in various food samples. Our results demonstrate that the optimization approach successfully improved the biosensor’s functionality, thus providing proof of concept for detecting chlorate in food samples. Our alternative biosensor offers a multitude of advantages, including ease of use, cost-effectiveness, on-site feasibility, high sensitivity, and a detection threshold that complies with the tolerance limits set by the public authorities of the European Union.

## 2. Materials and Methods

### 2.1. Chemicals Reagents and Strains

All solutions were prepared from ultrapure water obtained using a water purification system (PURELAB flex, Elga, Veolia). Sodium chlorate (NaClO_2_) used to determine the biosensor sensitivity was purchased from Acros Organics (Ref: #223222500). Glucose, glycerol, succinate, formate, and pyruvate were used at 0.2% as carbon sources. Trimethyl amine oxide (TMAO), fumarate, dimethyl sulfoxide (DMSO), and methionine sulfoxide (MetSO) were used at 7 mM as electron acceptors. Potassium nitrate (KNO_3_) was added at varying concentrations where mentioned.

The CH584 strain was constructed by transferring the ∆*msrP* Kan^R^ deletion mutant from the Keio collection strain (BW25113–JW1954) into strain CH183 via the standard P1 transduction procedure, and was checked via PCR. P1 phage transduction is a process by which the bacteriophage P1 transfers genetic material from one *E. coli* cell to another (horizontal gene transfer) [18].

The LL1290 strain was constructed as follows. An insert carrying the *msrP-lacZ-*∆*zinT* Kan^R^ genes was obtained after two PCRs using overlapping oligonucleotides. An *msrP-lacZ-msrQ’* fragment was amplified via PCR from CH183 strain using primers 350/847, and an *msrQ’-∆zinT* Kan^R^ fragment was obtained using 345/349 oligonucleotide pairs and the ∆*zinT* Kan^R^ strain as matrix (primer 345: cttcatattgccgacaaagtacgcgttatccctgcc; primer 349: cgtcatagtgatgagcgtgtctgtagctagcttgc; primer 847: ccacatccggcagggataacgcgtactttgtcggcaatatgaagttatttttgacaccagaccaactggtaatg; primer 350: ataaagcccatctctgccatcagaaacgg). The *msrP-lacZ-msrQ’* and *msrQ’-*∆*zinT Kan^R^* fragments were mixed and used as templates for a new PCR using the 350 and 349 oligonucleotide pair (primer 350: ataaagcccatctctgccatcagaaacgg). Replacement of the ∆*hiuH-msrPQ* Cm^R^ (LL996 strain) with the *msrP-lacZ-*∆*zinT* Kan^R^ fragment was performed as described by Datsenko and Wanner [19]. PCR analysis confirmed that the ∆*hiuH-msrPQ* Cm^R^ region had been exchanged with the *msrP-lacZ-*∆*zinT* Kan^R^ described above. P1 was used to move this allele into CH183, yielding the LL1290 strain.

The strains used in this study are given in Table 1.

### 2.2. Chlorate Induction Assays

Strains were grown overnight at 37 °C under anaerobic conditions in rich medium Lysogeny Broth (LB: Difco ref. 244620), in minimal M9 medium (Na_2_HPO_4_ (3,37 mM), KH_2_PO_4_ (2.2 mM), NaCl (0.86 mM), NH_4_Cl (0.936 mM), MgSO_4_ (1 mM)), or in minimal medium MA ((NH_4_)_2_SO_4_ (7.6 mM), KH_2_PO4 (33 mM), K_2_HPO_4_ (60.3 mM) and Na_3_C_6_H_5_O_7_ (1.9 mM)); they were supplied with a carbon source and electron acceptor where mentioned. Different concentrations of sodium chlorate (NaClO_3_) (0.5; 1; 2; 5; 10; 20 and 50 µM) and potassium nitrate (KNO_3_) (50; 500; 1000; 1500; 2000; 3000 µM) were tested. Growth under anaerobiosis was achieved by using 2 mL tubes full to the brim. Activities of β–galactosidase were measured as previously described [21]. This method involved the use of a lactose analog substrate, ortho-nitrophenyl-ß-D-galactopyranoside (ONPG). The enzyme hydrolyzed ONPG to produce nitrophenol, which could be quantified based on its yellow color and absorption at 420 nm. In a concise manner, the cells (200 µL of overnight culture) were resuspended in 800 µL of Z buffer (Na_2_HPO_4_ (0.06 M), NaH_2_PO_4_ (0.04 M), KCl (0.01 M), MgSO_4_ (0.001 M), ß-mercaptoethanol (0.05 M)), followed by the addition of one drop of 0.1% SDS and two drops of chloroform. The mixture was then vortexed for 10 s. The tubes were placed in a water bath at 28 °C for 5 min. The reaction was initiated by adding 200 µL of ONPG (4 mg/mL) and briefly agitated to ensure homogeneity. The reaction time was measured, and the reaction was stopped by adding 0.5 mL of 1 M NaCO_3_ when the yellow color appeared. After recording the optical density at 420 nm and 550 nm, Miller units were determined ((OD_420_ − (1.75 × OD_550_))/(culture time × culture volume × OD_600_).

### 2.3. Chlorate Analysis in Food Samples

The solid food samples tested were prepared with a laboratory mixer (Mixer MiniMix CC) using 50 g of food and 25 mL of ultrapure water mixed for 4 min at a speed of 4. Samples were then centrifuged at 16,000× *g* for 5 min to remove large particles. The food in powder form was suspended at 16% in ultrapure water. The samples were added to 2X M9 medium supplemented with glycerol 0.2% and 7 mM DMSO, and a chlorate induction assay was performed as mentioned above.

### 2.4. Statistical Analysis

Values are expressed as means with standard deviation (SD). Statistical analysis was performed using GraphPad Software Prism 8. Multiple-test analysis, with one per row, was performed to compare two groups at each concentration. Significance was assumed at * *p* < 0.05, ** *p* < 0.01, *** *p* < 0.001.

## 3. Results

### 3.1. M9 Minimal Medium Enhances Chlorate Detection and Reduces Nitrate Interference

**Figure 1:**
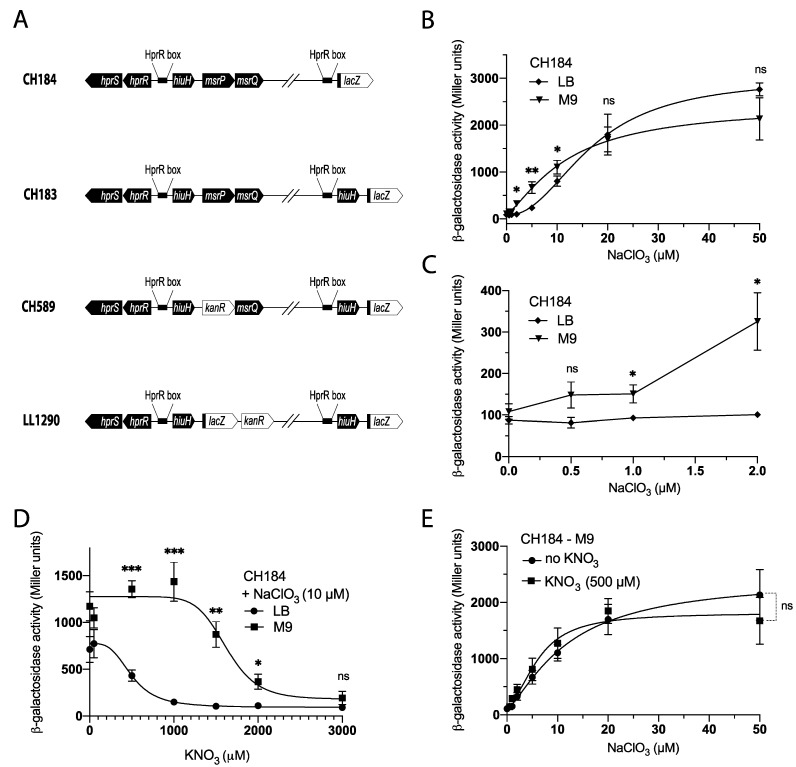
Improved chlorate detection with minimal medium and reduced nitrate interference. (**A**) Schematic representation of the chromosomal fusion of the strain used in this study. (**B**,**C**) Strain CH184 (*hiuH-lacZ*) was cultured overnight at 37 °C in LB or M9 glycerol supplemented, or not, with NaClO_3_ (0 to 50 μM), under anaerobic conditions, followed by ß-galactosidase assays. (**D**) Effect of nitrate (KNO_3_) on chlorate detection determined by following the *hiuH-lacZ* expression. Strain CH184 (*hiuH-lacZ*) was cultured overnight at 37 °C in LB or M9-glycerol supplemented with NaClO_3_ (10 µM) and KNO_3_ (0 to 3.000 µM), under anaerobic conditions, followed by ß-galactosidase assays. (**E**) Strain CH184 (*hiuH-lacZ*) was cultured overnight at 37 °C in M9-glycerol supplemented with NaClO_3_ (0 to 50 µM) and KNO_3_ (500 µM), under anaerobic conditions, followed by ß-galactosidase assays. Error bars indicate the standard deviation (*n* ≥ 3). Statistical analysis was performed using Student’s *t*-test (*** *p* ≤ 0.001; ** *p* ≤ 0.01; * *p* ≤ 0.05; ns, not significant).

In a previous study, we demonstrated that the cistron *hiuH*, which encodes a transthyretin-like periplasmic protein, and the *msrPQ* genes are part of the same operon [20]. The *hiuH-msrPQ* operon is induced in a HprSR-dependent manner by reactive chlorine species (RCS) [20,22]. Chlorate, an analogue of nitrate, is a substrate for nitrate reductases (NRs), which reduce it to the toxic chlorite (ClO_2_^−^) [15]. By examining the expression of the *hiuH-lacZ* fusion (strain CH184) (Figure 1A), we observed that the addition of chlorate, from 0 to 50 mM, to LB medium under anaerobic conditions led to an increase in ß-galactosidase activity (Figure 1B,C). Interestingly, when using minimal M9 medium instead of LB, we observed that the fusion responded better to lower NaClO_3_ concentrations (Figure 1B,C). This finding suggests that using M9 medium for chlorate detection is more efficient.

Nitrate (KNO_3_) is the physiological substrate of nitrate reductases (NRs), making it an effective competitor for chlorate reduction. We observed that the addition of nitrate during anaerobic growth in LB medium abolished the effect of chlorate on the induction of the *hiuH-lacZ* fusion (Figure 1D). Interestingly, using M9 medium reduced the inhibitory effect of nitrate on chlorate detection. We determined that the CH184 strain cultured in M9 medium was not affected by nitrate up to a concentration of 1000 μM (Figure 1D). Our results demonstrate that the *hiuH-lacZ* fusion is expressed similarly in the presence or absence of nitrate (500 μM) in the range of 0 to 50 μM chlorate when using M9-glycerol medium for chlorate detection (Figure 1E). These findings indicate that using M9-glycerol medium for chlorate detection minimizes the effect of nitrate with the CH184 strain.

### 3.2. Optimizing Chlorate Detection in E. coli Using Synthetic Biology

**Figure 2:**
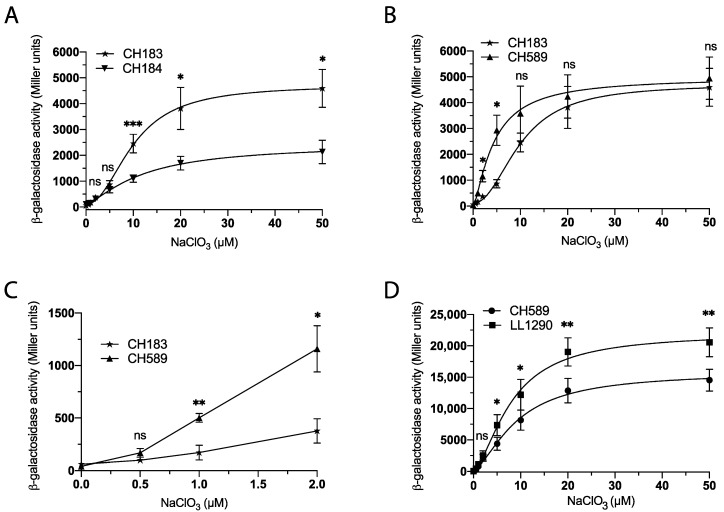
Enhancing Chlorate Sensing in *E. coli* through Synthetic Biology Techniques. (**A**) Using the *msrP-lacZ* instead of the *hiuH-lacZ* fusion. Strains CH184 (*hiuH-lacZ*) and CH183 (*msrP-lacZ*) were cultured overnight at 37 °C in M9-glycerol supplemented with NaClO_3_ (0 to 50 µM), under anaerobic conditions, followed by ß-galactosidase assays. (**B**,**C**) Using a ∆*msrP* strain increased *msrP-lacZ* fusion activity. Strains CH183 (*msrP-lacZ*) and CH589 (∆*msrP msrP-lacZ*) were cultured overnight at 37 °C in M9-glycerol supplemented with NaClO_3_ (0 to 50 µM), under anaerobic conditions, followed by ß-galactosidase assays. (**D**) Two msrP-lacZ fusions in the chromosome. Strains CH589 (∆*msrP msrP-lacZ*) and LL1290 (∆*msrP::msrP-lacZ msrP-lacZ*) were cultured overnight at 37 °C in M9-glycerol supplemented with DMSO (7 mM) in the presence of NaClO_3_ (0 to 50 µM), under anaerobic conditions, followed by ß-galactosidase assays. Error bars indicate the standard deviation (*n* ≥ 3). Statistical analysis was performed using Student’s *t*-test (*** *p* ≤ 0.001; ** *p* ≤ 0.01; * *p* ≤ 0.05; ns, not significant).

Our next objective was to modify the genome of *E. coli* to determine the best combination of mutation and fusion for chlorate detection. First, we assessed the variation in the induction levels of *hiuH-lacZ* (CH184) and *msrP-lacZ* (CH183) (Figure 1A) fusions in response to chlorate concentration (0 to 50 μM). Our results show a significant (approximately 2-fold) increase in ß-galactosidase activity in the *msrP-lacZ* (CH183) fusion compared to the *hiuH-lacZ* (CH184) fusion (Figure 2A). Consequently, the *msrP-lacZ* fusion is a better choice for chlorate detection since it exhibits a stronger response than the *hiuH-lacZ* fusion. Secondly, we sought to further investigate the role of the presence of MsrP in chlorate detection. Our previous study demonstrated a negative feedback effect of MsrP on the expression of the *hiuH-msrPQ* operon, which was attributed to the methionine redox control process that activates the HprS sensor [20]. We examined the induction of the *msrP-lacZ* fusion in the presence (CH183) or absence (CH589) of MsrP. Our results indicate that strain CH589, which lacks MsrP, exhibited higher ß-galactosidase activity compared to CH183, particularly in the 1 to 5 μM chlorate concentration range (Figure 2B,C). This result shows that the deletion of *msrP* is a promising strategy for enhancing the sensitivity of the biosensor for chlorate detection.

Thirdly, in order to further optimize our biosensor for chlorate detection, we introduced an *msrP-lacZ* fusion in place of the *msrP* gene in the CH183 strain. This modification leads to engineering of the LL1290 strain (Figure 1A), which lacks MsrP and contains two copies of the *msrP-lacZ* fusion. Our results showed that the LL1290 strain exhibits increased ß-galactosidase activity compared to CH589, particularly in response to chlorate concentrations ranging from 5 to 50 μM (Figure 2D). Of note, this experiment was conducted in the presence of DMSO, which increased the detection efficiency of chlorate and whose usefulness is explained in the following paragraph.

### 3.3. Optimizing Chlorate Detection in E. coli by Modifying the Culture Medium

**Figure 3:**
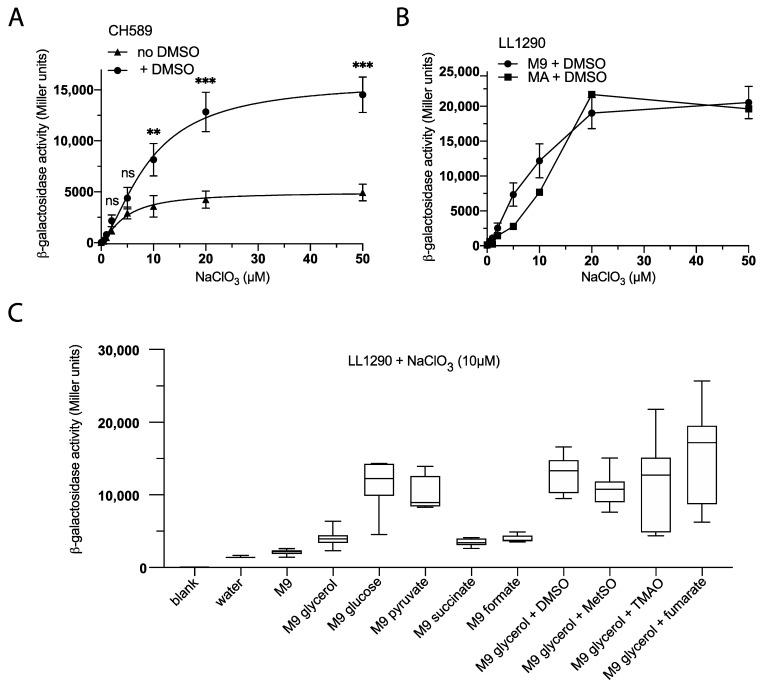
Enhancing Chlorate Detection in *E. coli* via Cultivation Medium Modification. (**A**) Adding DMSO to M9-glycerol increased *msrP-lacZ* fusion activity. Strain CH589 (∆*msrP msrP-lacZ*) was cultured overnight at 37 °C in M9-glycerol supplemented, or not, with DMSO (7 mM) in the presence of NaClO_3_ (0 to 50 µM), under anaerobic conditions, followed by ß-galactosidase assays. (**B**) Strain LL1290 (∆*msrP::msrP-lacZ msrP-lacZ*) was cultured overnight at 37 °C in M9-glycerol or MA-glycerol supplemented with DMSO (7 mM) in the presence of NaClO_3_ (0 to 50 µM), under anaerobic conditions, followed by ß-galactosidase assays. (**C**) Strain LL1290 (∆*msrP::msrP-lacZ msrP-lacZ*) was cultured overnight at 37 °C in M9 supplemented with different carbon sources and electron acceptors in the presence of NaClO_3_ (10 µM), under anaerobic conditions, followed by ß-galactosidase assays. Error bars indicate the standard deviation (*n* ≥ 3). Statistical analysis was performed using Student’s *t*-test (*** *p* ≤ 0.001; ** *p* ≤ 0.01; ns, not significant).

In order to improve the efficiency of chlorate detection by *E. coli*, we investigated the impact of modifying the culture medium by adding different carbon sources and electron acceptors. First, using the CH589 strain, we observed that the addition of the electron acceptor dimethyl-sulfoxide (DMSO) to the M9 medium resulted in a significant increase in ß-galactosidase activity. Specifically, we found that the *msrP-lacZ* fusion showed a three-fold increase in activity in the presence of DMSO, particularly in response to chlorate concentrations ranging from 10 to 50 μM (Figure 3A). These findings suggest that the addition of DMSO to the culture medium may represent a promising strategy for enhancing the sensitivity of biosensors for chlorate detection. We next investigated the impact of the culture medium on the biosensor in response to NaClO_3_ concentration. Specifically, we cultured LL1290 strains in M9 and MA minimal media supplemented with DMSO and NaClO_3_ concentrations ranging from 0 to 60 µM. Interestingly, we found that the ß-Galactosidase activities were similar regardless of the culture medium used (Figure 3B). These results indicate that the biosensor can be used in any minimal culture medium, providing greater flexibility for the detection of chlorate.

Finally, we assessed the impact of various carbon and electron acceptor sources on the expression of the reporter in LL1290 strains cultured in an M9 minimal medium supplemented with 10 µM NaClO_3_. Our investigation included the use of different carbon sources, such as glycerol, glucose, pyruvate, succinate, and formate, as well as electron acceptors, such as DMSO, methionine sulfoxide (MetSO), trimethylamine N-oxide (TMAO), and fumarate. The results indicate that chlorate, at a concentration of 10 µM, could be detected in all tested media, including water (Figure 3C). However, the presence of a carbon source, particularly glucose or pyruvate, led to significantly improved detection results. Moreover, we found that any tested electron acceptor sources increased the sensitivity of the chlorate biosensor (Figure 3C). These observations suggest that the choice of carbon and electron acceptor sources significantly affects the detection of chlorate and must be taken into account when designing detection strategies.

### 3.4. Validation of a Bacterial Biosensor for the Detection of Chlorate in Food

After optimizing the biosensor, we sought to evaluate its performance on different food samples. To assess the specificity and sensitivity of the biosensor, we selected a range of food commodities, including tap and mineral water, vegetables, fruits, cereals, and milk.

**Figure 4:**
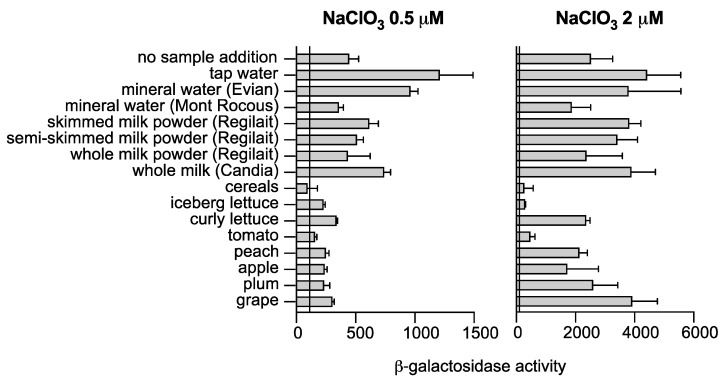
Detection of chlorate in food using a bacterial biosensor. Strain LL1290 (∆*msrP::msrP-lacZ msrP-lacZ*) was cultured overnight at 37 °C in M9 supplemented with DMSO (7 mM) under anaerobic conditions in presence of the food sample containing either 0.5 or 2 µM NaClO_3_. Subsequently, ß-galactosidase assays were conducted, with the error bars representing the standard deviation (*n* ≥ 3).

The optimized version of the biosensor, strain LL1290, was grown in M9-glycerol medium supplemented with DMSO under anaerobic conditions in the presence of the food sample. We measured the activity of ß-galactosidase in each sample after spiking it with varying concentrations of chlorate, since no endogenous chlorate contamination was detected. Our study shows that the biosensor is capable of detecting chlorate contamination in various food and water samples, including tap and mineral water; curly and iceberg lettuce; grape; plum; apple; peach; tomato; and skimmed, semi-skimmed, and whole milk powder, with a minimum threshold of 0.5 µM (Figure 4, left panel). Additionally, the biosensor can detect chlorate in cereals with a threshold of 2 µM (Figure 4, right panel). Our results demonstrate the versatility of the biosensor in detecting chlorate contamination in a variety of food and water samples.

## 4. Discussion

Synthetic biology has made it possible to genetically engineer bacteria and create bacterial biosensors that can detect a specific molecule or chemical through intrinsic regulatory mechanisms. Typically, these biosensors involve a sensing module and a reporting module that generates a readily detectable output signal [23,24,25]. For the chlorate biosensor we present in this study, we used the bacteria *Escherichia coli*. The sensing module comprises an HprSR two-component system, which is triggered by the oxidation of methionine residues located in the periplasmic domain of HprS [20,26]. Meanwhile, the reporting module is represented by the expression level of the *hiuH-msrPQ* operon. Notably, this biosensor is specifically designed for chlorate detection and must be used under anaerobic conditions due to its reliance on nitrate reductase activities [15]. By comparing sample analyses with the biosensor under anaerobic and aerobic conditions, we can establish the specificity of chlorate presence and differentiate it from other chlorinated derivatives such as HOCl. Specifically, only chlorate will yield a positive test result under anaerobic conditions and a negative test result under aerobic conditions. In contrast, other chlorinated derivatives will produce positive test results under both conditions. We enhanced the efficiency of the biosensor by optimizing the growth conditions, including the use of a minimal medium supplemented with an electron acceptor. This optimization was crucial in eliminating the antagonistic impact of nitrate, which could potentially be present in the samples being analyzed. Other molecules besides nitrate could probably interfere with our measurements. Specifically, we observed that elevated levels of TMAO can inhibit the production of MsrP in the presence of chlorate [15]. Given that TMAO is naturally found in seafood [27,28], it may pose a limitation to our technology. Additionally, we can speculate on the potential negative effects of molecules capable of scavenging chlorite before it activates the HprSR pathway. An exhaustive interference study is currently underway to determine compounds encountered in food samples that are capable of interfering with chlorate measurement. Additionally, the use of two copies of the reporter fusions and the deletion of the *msrP* gene from the genome allowed us to amplify the response. Through this optimization, we were able to detect chlorate in concentrations in the micromolar range, meeting the expectations of industry and regulatory authorities. This approach allowed us to assess the performance of the biosensor in detecting chlorate in different food samples. The tolerance threshold for chlorate in drinking water is set at 3 µM, and is increased to 8.4 µM in the case of water treated with chlorinated derivative compounds for disinfection purposes. The maximum limits applicable to food products in the European Union (REGULATION 2020/749 amending Annex III (EC) No 396/2005) depend on the specific products, and range from 0.05 to 0.7 mg of chlorate/kg of food. This biosensor is capable of detecting the presence of chlorate with a detection threshold of 0.5 µM in water. Our results also demonstrate that this threshold can vary for different samples, reaching 2 µM or higher for certain foods. A comprehensive study of relevant food items will be necessary in the future to address this issue effectively. Our findings confirm that our optimization technique effectively enhanced the biosensor’s performance and validated its use in the detection of chlorate in food samples. This serves as proof of concept of the biosensor’s ability to detect chlorate in a variety of food products.

## Figures and Tables

**Table 1:** Strains used in this study.

Strain	Genotype	Source
CH184	MG1655 *hiuH-lacZ* (in *lac* locus)	El Hajj et al. (2022) [20]
CH183	MG1655 *msrP-lacZ* (in *lac* locus)	El Hajj et al. (2022) [20]
CH589	MG1655 ∆*msrP* Kan^R^ *msrP-lacZ* (in *lac* locus)	This study
LL1290	MG1655 *msrP-lacZ* Kan^R^ (in *hiuH-msrPQ* locus) *msrP-lacZ* (in *lac* locus)	This study

## Data Availability

Not applicable.

## Patents

This work is protected by an international patent under filing number PCTEP2023060172.
